# Benzene oxygenation and oxidation by the peroxygenase of *Agrocybe aegerita*

**DOI:** 10.1186/2191-0855-3-5

**Published:** 2013-01-17

**Authors:** Alexander Karich, Martin Kluge, René Ullrich, Martin Hofrichter

**Affiliations:** 1 Department of Bio- and Environmental Sciences, International Graduate School of Zittau, Markt 23, 02763, Zittau, Germany

**Keywords:** Peroxidase, P450, Epoxide, Oxepin, Oxygen transfer

## Abstract

Aromatic peroxygenase (APO) is an extracellular enzyme produced by the agaric basidiomycete *Agrocybe aegerita* that catalyzes diverse peroxide-dependent oxyfunctionalization reactions. Here we describe the oxygenation of the unactivated aromatic ring of benzene with hydrogen peroxide as co-substrate. The optimum pH of the reaction was around 7 and it proceeded via an initial epoxide intermediate that re-aromatized in aqueous solution to form phenol. Identity of the epoxide intermediate as benzene oxide was proved by a freshly prepared authentic standard using GC-MS and LC-MS analyses. Second and third [per]oxygenation was also observed and resulted in the formation of further hydroxylation and following [per]oxidation products: hydroquinone and *p*-benzoquinone, catechol and *o*-benzoquinone as well as 1,2,4-trihydroxybenzene and hydroxy-*p*-benzoquinone, respectively. Using H_2_^18^O_2_ as co-substrate and ascorbic acid as radical scavenger, inhibiting the formation of peroxidation products (e.g., *p*-benzoquinone), the origin of the oxygen atom incorporated into benzene or phenol was proved to be the peroxide. Apparent enzyme kinetic constants (k_cat_, K_m_) for the peroxygenation of benzene were estimated to be around 8 s^-1^ and 3.6 mM. These results raise the possibility that peroxygenases may be useful for enzymatic syntheses of hydroxylated benzene derivatives under mild conditions.

## Introduction

Benzene is a poorly activated aromatic hydrocarbon that naturally occurs in crude oil. Worldwide it plays an important role as a most basic petrochemical (Fruscella 2000). Although it is not a common compound in the biosphere, some bacteria are able to metabolize benzene as sole carbon source. *Pseudomonas putida* converts it with a Rieske-type benzene 1,2-dioxygenase (EC 1.14.12.3) to benzene *cis*-dihydrodiol, which is also a side reaction catalyzed by toluene dioxygenase (EC 1.14.12.11) from the same bacterium. The former enzyme consists of three components, a flavoprotein reductase, a ferredoxin and a catalytic iron-sulfur protein (Bagnéris et al. 2005). Other bacteria produce soluble di-iron monooxygenases, for example, the four-component enzymes found under EC 1.14.13.-, some of which can oxidize benzene and other aromatics. Soluble methane monooxygenase (sMMO; EC 1.14.13.25) is an example of such a monooxygenase with exceptionally broad substrate spectrum (Colby et al. 1977; Leahy et al. 2003; Tao et al. 2004; Zhou et al. 1999). In eukaryotes including humans, benzene is hydroxylated by cytochrome P450 monooxygenases (EC 1.14.14.1) using a similar reaction mechanism as the di-iron enzymes (Froland et al. 1992; Green and Dalton 1989; Seaton et al. 1994; Tamie and Rui-Sheng 1994).

A functional hybrid of monooxygenases and peroxidases is represented by a just recently accepted group of heme-thiolate proteins referred to as aromatic or unspecific peroxygenases (EC 1.11.2.1) (Hofrichter et al. 2010). The most detailedly studied member of this group is the *Agrocybe aegerita* aromatic peroxygenase (*Aae*APO), which is secreted by an agaric fungus, the Black poplar mushroom. *Aae*APO was found to catalyze a variety of oxyfunctionalization reactions such as hydroxylation of saturated hydrocarbons (Kluge et al. 2012; Peter et al. 2011; Ullrich and Hofrichter 2005), epoxidation of unsaturated hydrocarbons (Kluge et al. 2009; Kluge et al. 2012), heterocyclic *N*-oxidation (Ullrich et al. 2008), sulfoxidation (Aranda et al. 2009) and *O*-dealkylation (ether cleavages (Kinne et al. 2009b)). In contrast to the distantly related chloroperoxidase from *Caldariomyces fumago* (CPO; EC 1.11.1.10), APOs are able to peroxygenate/hydroxylate aromatic hydrocarbons, e.g. of toluene and naphthalene (Hofrichter et al. 2010). For the latter reaction, an epoxide intermediate was identified and the origin of the incorporated oxygen was proved to be the peroxide (Kluge et al. 2009). Earlier findings have given first indication that *Aae*APO is also capable of oxidizing benzene but the underlying reaction has not been studied in detail, so far.

Whereas in all benzene oxygenations catalyzed by monooxygenases, the electron donor is NAD(P)H and the oxygen donor molecular oxygen (O_2_), unspecific peroxygenases use hydrogen peroxide (H_2_O_2_) as oxygen donor and electron acceptor. This and the fact that peroxygenases are stable extracellular enzymes make them an interesting biocatalytic tool both for basic studies on oxygen transfer reactions and biotechnological applications. Here we describe the peroxygenase-catalyzed, multiple hydroxylation of benzene that proceeds via the initial formation of benzene oxide.

## Materials and methods

### Chemicals and enzyme preparation

All chemicals used were purchased from Sigma-Aldrich (Munich, Germany) with highest purity available except H_2_^18^O_2_, which was obtained from Icon Isotopes (Summit, NJ) and benzene oxide which was synthetized by a method similar to that used by Platt and Oesch (1977). In short, dibromination of 1,4-cyclohexadien with elemental Br_2_ in chloroform at 0°C led to 4,5-dibromocyclohexene (van Tamelen 1955). The oxidation of 4,5-dibromocyclohexene leading to 4,5-dibromocyclohexene oxide was realized with *m*-chloroperoxybenzoic acid (*m*CPBA) (Gillard et al. 1991). Benzene oxide was formed by further dehydrobromination with diazobicyclo [5.4.0] undec-7-ene (DBU) mixed with acetonitrile (1:4). The 70 eV mass spectra of the synthetized standard were largely identical to the mass spectra issued by Lovern et al. (1997).

*A*. *aegerita* aromatic peroxygenase (*Aae*APO) was purified from cultures of *A*. *aegerita* strain TM-A1 (deposited at the Leibnitz institute DSMZ - German Collection of Microorganisms and Cell Cultures; collection number DSM 22459) by several steps of fast protein liquid chromatography (FPLC) as described previously (Ullrich et al. 2004). The final enzyme preparation had a specific activity for veratryl alcohol of 83.7 U mg^-1^ and an Rz_(420/277)_ value (*Reinheitszahl*) of 1.3.

### Enzymatic reactions

One-pot reaction mixtures contained 20 mM potassium phosphate buffer of pH 3, 4.5, 6, 7.5, 9 or 10.5 for evaluation, and pH 7 for any other reaction setup. Substrate concentrations were 22.5 mM for benzene and 0.5 mM for phenol. For kinetic studies, benzene concentration was varied between 0.32 mM and 5 mM. Every reaction mixture contained 1.56 μM *Aae*APO; for kinetic studies, this value was halved. The hydrogen peroxide (H_2_O_2_) concentration routinely used in the reaction mixtures was 1 mM and for kinetic studies 6 mM (as a sum). In the first case, it was continuously added via a syringe pump over 30 min (1 μmol h^-1^). In the course of oxygen incorporation studies, benzene oxide experiments as well as kinetic studies, H_2_O_2_ was quickly added with a pipette (one-time addition of 1 mM). In kinetic studies, a mixture of H_2_O_2_ and benzene was used to start the reaction that was carried out in the presence of 5% (v/v) acetonitrile. The final concentration of added ascorbic acid for the oxygen incorporation and kinetic studies was 4 mM and 8 mM, respectively. All reactions were performed in HPLC vials in a final volume of 0.5 ml.

### High performance liquid chromatography (HPLC) and liquid chromatography – mass spectrometry (LC-MS)

HPLC analyses were performed with a 1200 Series Agilent system (Waldbronn, Germany) with diode-array UV–vis detection. For reversed phase chromatographic separation, a Luna® 5 μm C18 (2) 100 Å column (150 × 2 mm, Phenomenex, Aschaffenburg, Germany) was used. The mobile phase consisted of (A) ammonium formate buffer (0.1%; pH 3.6), and (B) acetonitrile. For the standard analysis, 5% B was held for 5 min, then raised to 100% B within 17 min and held again for 3 min. The analysis of samples containing benzene oxide was performed differently, i.e. by holding 20% B for 2 min, then raising up to 95% B within 7 min and holding this concentration for 1.5 min. The spectra of eluting substances were recorded between 200 and 500 nm.

After separation, the samples were also analyzed with an ion trap mass spectrometer (6300 Series, Agilent, Waldbronn, Germany). Ionization was achieved by atmospheric pressure chemical ionization (APCI) in the negative mode. Mass spectra were recorded in the range from 50 to 180 m/z.

### Gas chromatography – mass spectrometry (GC-MS)

For the gas chromatographic detection of benzene oxide, the reaction mixture (0.5 ml) was extracted with dichloromethane (100 μl) and 1 μL of the extract was analyzed with a GC-MS system (Agilent 6890 GC, 5973 MS, Waldbronn, Germany) equipped with a BPX5 capillary column (30 m × 0.25 mm × 0.25 μm FT; SGE Europe Ltd., Buckinghamshire, UK). The sample was injected with a split ratio of 5:1 at 250°C. Initial oven temperature of 65°C was held for 1 min and then increased to 190°C at a rate of 45°C min^-1^. Helium carrier gas was supplied at a rate of 1 ml min^-1^.

### Further analytical methods

To quantify the reaction products, calibration curves were prepared using authentic standards and the HPLC separation method described above. LC-MS analysis of both hydroquinone and *p*-benzoquinone gave anions of 108 *m*/*z* (the formation of the hydroquinone anion was already reported previously by (Letzel et al. 2001)). Catechol gave a 109 *m*/*z* anion also previously observed (Albarran et al. 2010). As we did, the authors observed the same weight ion for the structurally related benzoquinones.

^18^O-Labeled hydrogen peroxide (H_2_^18^O_2_) was used as cosubstrate to prove the origin of the transferred oxygen. Peroxygenase-catalyzed incorporation of ^18^O yielded isotopologue ions shifted by 2 *m*/*z* per incorporated oxygen.

Formation of the major products, i.e. the amounts of phenol, hydroquinone, catechol and *p*-benzoquinone formed during the reaction, was used for the estimation of kinetic data. Reaction conditions were the same as described above except that the benzene concentration was varied between 310 μM and 5,000 μM and a smaller amount of *Aae*APO (0.78 μM) was used. Reactions were performed in triplicate, mixed with 2 parts of HPLC-mobile phase (A) and injected, after 45 s, into the HPLC system. The concentration of products was determined by HPLC as described above. Michaelis-Menten constant (K_m_) and catalytic constant (k_cat_) were obtained by nonlinear regression applying the Michaelis-Menten model in the Anemona program (Hernández and Ruiz 1998).

## Results

### Products of benzene oxidation and effect of pH

**Figure 1:**
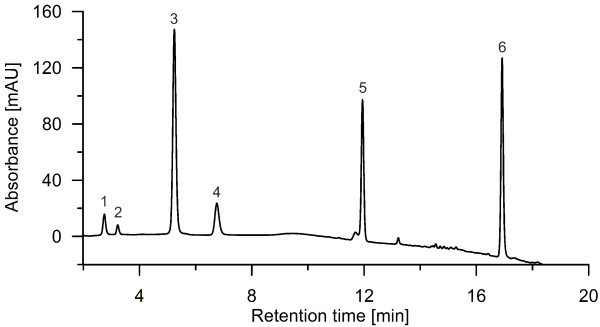
**Reversed phase HPLC elution profile (recorded at 220 nm) of benzene hydroxylation catalyzed by *Aae*APO at pH 7.5 with eluting products hydroquinone (1), hydroxy-*p-*benzoquinone (2), *p*-benzoquinone (3), catechol (4), and phenol (5); peak (6) refers to the substrate benzene.**

**Figure 2:**
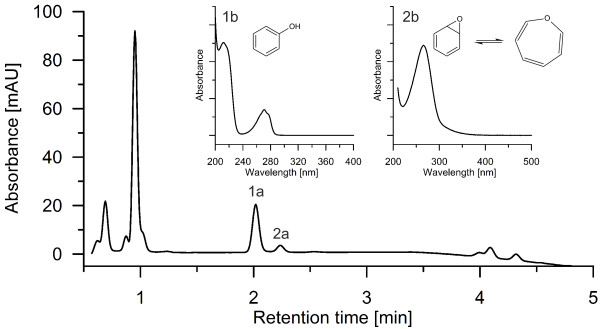
**Reversed phase HPLC elution profile (recorded at 220 nm) of a benzene sample treated with *Aae*APO: phenol (1a) and benzene oxide (2a).** The insets show the UV–vis spectra of phenol (1b), and benzene oxide (2b).

**Figure 3:**
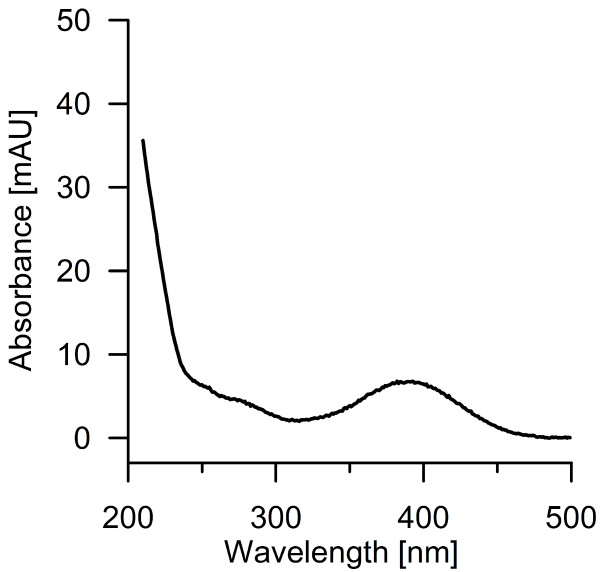
**UV/vis-spectrum of a short-lived benzene oxidation product found at low pH (<4),probably representing *o-*benzoquinone (Albarran et al.**^2010^**).**

**Figure 4:**
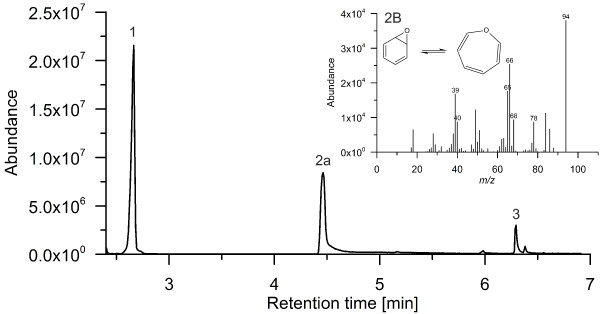
**Gas chromatogram of a benzene sample treated with *Aae*APO: benzene (1), benzene oxide (2a), and phenol (3).** The inset (2b) shows the 70 eV mass spectrum of benzene oxide obtained by LC-MS/MS.

By analyzing the products formed by *Aae*APO from benzene with reversed phase HPLC, a spectrum of different hydroxylation and oxidation products was observed (Figure 1). Most important, the detection of phenol proved the difficult to accomplish [per] oxygenation/hydroxylation of benzene (Figure 2). Second hydroxylation was confirmed by detecting hydroquinone and catechol as well as their oxidation products *p*-benzoquinone (Figure 1) and *o*-benzoquinone (Figure 3), respectively. Figure 3 shows the UV–vis spectrum of a short-lived product found at low pH that resembles the spectrum of *o*-benzoquinone published by (Albarran et al. 2010). Third hydroxylation was proved by the detection of traces of 1,2,4-trihydroxybenzene and hydroxy-*p*-benzoquinone (Figure 1). All identified products co-eluted and shared their UV–vis spectra with authentic standards. Since there was no authentic benzene oxide standard available to prove the postulated initial epoxidation, benzene oxide was synthesized according to the method reported by Platt and Oesch (1977). Using this standard, the formation of benzene oxide was confirmed both by HPLC and GC-MS analyses (Figures 2 and 4).

Coupling and polymerization of phenolic products formed was observed in all reactions without ascorbic acid and led to numerous small peaks eluting late from the HPLC column with unincisive UV–vis and mass spectra (data not shown).

**Figure 5:**
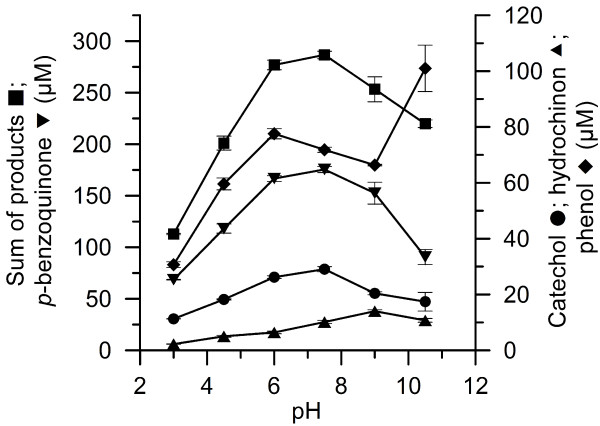
**Effect of pH on the concentration of products formed in the course of benzene oxygenation/hydroxylation by *Aae*APO.**

Since benzene oxide, *o*-benzoquinone, trihydroxybenzene and hydroxyquinones were found only in traces (<3 μM), just the concentrations of phenol, hydroquinone, catechol and *p*-benzoquinone were used to evaluate the effect of pH on the catalytic performance. Altogether, the formation of these major products (sum of them) showed a pH optimum around 7.5, although there were individual deviations from that for phenol and hydroquinone, in the case of which highest amounts were detectable at pH 10.5 and 9, respectively (Figure 5).

### Incorporation of oxygen

^18^O-Labeled hydrogen peroxide (H_2_^18^O_2_) was used as probe to study the incorporation of oxygen into benzene and the isotopic distribution of ^18^O in the products. Reactions were carried out with or without ascorbic acid as phenoxyl radical scavenger and analyzed by LC-MS. Mass spectra of catechol and *p*-benzoquinone but not of phenol were obtained by the LC-MS method used (Table 1). In the absence of ascorbic acid, we observed a substantial incorporation of ^18^O originating from H_2_^18^O_2_ in *p*-benzoquinone but not in catechol. In the presence of ascorbic acid, peroxygenation was detectable both for hydroquinone and catechol. The ratio of incorporated oxygen coming from the peroxide was higher in *p*-benzoquinone (or hydroquinone) than in catechol. The same trend was observed when phenol was used as substrate (Tab. 1); yet, the ratios of incorporated oxygen coming from hydrogen peroxide in both hydroquinone and catechol approached 100% when ascorbic acid was used.

**Table 1:** **Origin of oxygen in the hydroxylation and oxidation products of benzene and phenol formed by *Aae*APO in the presence of H**_**2**_^**18**^**O**_**2**_

**Substrate**	**Oxygen atoms in hydroquinone [rel.%]**			**Oxygen atoms in *p***-**benzoquinone [rel.%]**			**Oxygen atoms in catechol [rel.%]**		
	^**16**^**O**/^**16**^**O**	^**16**^**O**/^**18**^**O**	^**18**^**O**/^**18**^**O**	^**16**^**O**/^**16**^**O**	^**16**^**O**/^**18**^**O**	^**18**^**O**/^**18**^**O**	^**16**^**O**/^**16**^**O**	^**16**^**O**/^**18**^**O**	^**18**^**O**/^**18**^**O**
Benzene	n.d.^§^	n.d.^§^	n.d.^§^	20	55	25	100	0	0
Benzene^#^	9	25	66	n.d.^§ §^	n.d.^§ §^	n.d.^§ §^	18	31	51
Phenol	42	58	0	42	58	0	100	0	0
Phenol^#^	3	97	0	n.d.^§ §^	n.d.^§ §^	n.d^§§^	6	94	0

^#^ in presence of ascorbic acid (4 mM).

^§^ n.d. – not detectable, the concentration was too low to give a viable LC-MS signal.

^§§^ n.d. – not detected in the presence of ascorbic acid.

### Kinetic parameters for benzene hydroxylation

Several facts hampered the exact determination of kinetic constants for benzene oxidation by *Aae*APO. The HPLC method used required a preparation time of about 45 sec, which made a real-time/time-resolved analysis impossible. In addition, benzene is a highly volatile compound (tending to “crawl” along the glass and disappear that way from the reaction mixture), which made its decrease nearly unfeasible to determine exactly. Thus the formation of products had to be used for the estimation of kinetic data. However, when benzene is first converted into phenol, it immediately undergoes oxidation into further products, since phenol is a far better substrate than benzene.

**Figure 6:**
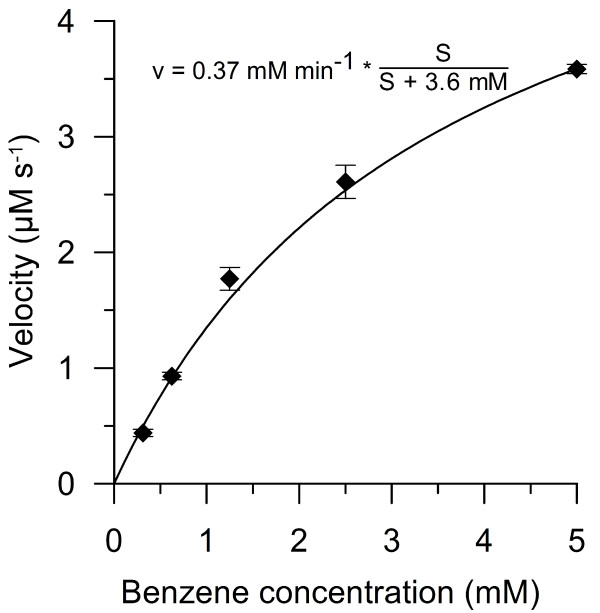
**Conversion rates for benzene obtained with 0.78 μM *Aae*APO in the presence of 6 mM hydrogen peroxide as a function of benzene concentration.** The conversion of benzene was followed by detecting the concentrations of phenol, hydroquinone and catechol (sum of them) by HPLC.

Albeit the evaluation of benzene oxidation using the amount of different products formed describes an overall reaction that includes several hydroxylation steps, this approach was found to be suitable to approximate the order of magnitude of kinetic values. Thus using the sum of the products phenol, hydroquinone, catechol and *p*-benzoquinone as measure, apparent kinetic constants (k_cat_, K_m_) for benzene hydroxylation were estimated to be 8 sec^-1^ and 3.6 mM (Figure 6), and the resulting catalytic efficiency 2.2 x 10^3^ M^-1^ s^-1^.

## Discussion

The variety of products formed in the course of benzene oxidation by *Aae*APO reflects its ability to hydroxylate, in addition to benzene, also phenol and dihydroxybenzenes, which further undergo oxidation into quinones. It is reasonable that phenol represents a better substrate than the little activated benzene, because of the higher electron density at the aromatic ring. Thus phenol is oxidized by *Aae*APO leading to peroxygenation/hydroxylation at *para*- or *ortho*-position to form hydroquinone or catechol. Since *p*-benzoquinone is the oxidation product of hydroquinone formed by *Aae*APO in the peroxidase mode, the obtained data give evidence that *para*-hydroxylation is favored over *ortho*-hydroxylation. Formation of 1,2,4-trihydroxybenzene resulting from the hydroxylation either of catechol or hydroquinone led to the conclusion that the reactions catalyzed by *Aae*APO are, in contrast to toluene monooxygenase, more similar to the benzene metabolism by cytochrome P450s as proposed in previous reports (Snyder et al. 1993; Tao et al. 2004).

The pH optimum for the overall reaction was found to be around pH 7. That information fits well to earlier results concerning the pH optima of hydroxylations catalyzed by *Aae*APO (Ullrich and Hofrichter 2005; Ullrich et al. 2004). The finding that the highest concentration of phenol was detectable at pH 10.5 is a notable fact. A plausible explanation for that may be the pK_a_ of phenol (9.9). At a pH value above the pK_a_, a large portion (>50%) of phenol is present as phenolate ion. The polar phenolate may not well be directed into the heme channel of the enzyme, which is lined with many non-polar phenylalanine residues (Piontek et al. 2010). Hence phenol formed was hardly converted at a pH above its pK_a_ and thus accumulated.

The analytical data of Figures 2 and 4 clearly show that the *Aae*APO-catalyzed hydroxylation of benzene to phenol proceeds via a transient epoxide intermediate (benzene oxide that is in equilibrium with oxepin). This finding is in accordance with the results of naphthalene hydroxylation by *Aae*APO, in the course of which also an unstable epoxide (naphthalene 1,2-oxide) was formed as first product (Kluge et al. 2009). Benzene hydroxylation by cytochrome P450 monooxygenases was reported to proceed via benzene oxide as well, but furthermore the direct hydroxylation to phenol or the oxygenation via a ketone intermediate (cyclohexenone) was proposed (Bathelt et al. 2008; de Visser and Shaik 2003). Since we did not find indications for the formation of the latter both intermediates, we assume that epoxidation is the major route of benzene peroxygenation by *Aae*APO (compare also Barková et al. (2011) Kluge et al. (2009)).

**Figure 7:**
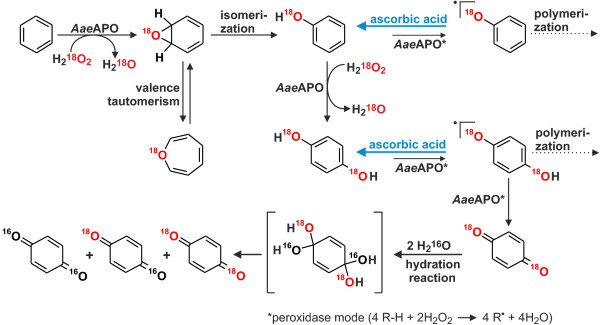
**Hypothetic scheme for the epoxidation/hydroxlyation and further oxidation of benzene and its following products phenol and hydroquinone by *Aae*APO; in the same way, catechol may be oxidized to *o*-benzoquinone.**

The origin of incorporated oxygen in true peroxygenase reactions must be the peroxide. At first glance, this did not seemingly correspond well with the data found here using H_2_^18^O_2_ as cosubstrate during benzene and phenol oxidation (Table 1). A plausible explanation for these results may be the ability of quinones to exchange their oxygen atoms with water (Fesenko and Gragerov 1955). The fact that *o*-benzoquinone is known to exchange its oxygen much faster with water than *p*-benzoquinone does, is reflected in the differences between the oxygen isotope distribution in *p*-benzoquinone (or hydroquinone) and catechol (Tab. 1). The formation of quinones was prevented by adding ascorbic acid to the reaction mixture, which led, in all cases, to an increase of the “heavy” oxygen isotope (^18^O) in both hydroquinone and catechol (Tab. 1). Yet ascorbic acid did not inhibit the formation of quinones but instantly reduced them. Hence there was still a little moiety of the oxidized form that underwent oxygen exchange. That is why no product with 100% ^18^O-incorporation was observed. Since ascorbic acid furthermore “blocks” the full peroxidase-mode of *Aae*APO (one-electron oxidation) by reducing formed phenoxyl radicals, there was no other possibility for the oxidation of phenol than the peroxygenation (Kinne et al. 2009a). By blocking the peroxidase-mode, also the further coupling of phenoxyl radicals was prevented (Figures 1 and 7).

The obtained kinetic constants for benzene hydroxylation by *Aae*APO cannot be regarded as simply “apparent” with the applied reaction setup. That is because the analysis of the reaction products was realized by HPLC using the sum of four different products and therefore could not be run as real-time analysis. Different reactions occurred within the one and only reaction pot, e.g. the further hydroxylation of phenol. Nevertheless, the order of magnitude of the obtained kinetic constants may be helpful in evaluating the reaction. So the calculated catalytic efficiency (~2 × 10^3^ M^-1^ s^-1^) for benzene peroxygenation by *Aae*APO is rather low and in the same order of magnitude as the oxidation of pyridine that was also difficult to oxidize by *Aae*APO (Ullrich et al. 2008). Hanioka described an apparent K_m_ for benzene hydroxylation by a human cytochrome P450 monooxygenase that was with about 10 mM three times higher than the value reported herein but still in the same order of magnitude (Hanioka et al. 2010).

The oxygenation/hydroxylation of benzene activates the molecule and thereby increases its biological accessibility by raising the solubility in water and the electron density of the ring (that is important in terms of biodegradability). These effects apply to the second and third hydroxylation as well. Aerobic degradation of aromatic compounds via ring-cleavage of catechol derivatives leading to the formation of *ciscis*-muconic acids and eventually of succinate and acetyl-CoA is a well-known metabolic pathway in many bacteria, and similar pathways are also present in fungi (Hofrichter et al. 1992; Jindrová et al. 2002; Sala-trepat and Evans 1971). Nevertheless benzene oxidation by a heme peroxidase/peroxygenase is reported herein for the first time. The first hydroxylation is the toughest-to-catalyze step and essential for the further degradation of the aromatic ring. Whether this reaction is involved in the co-metabolic oxidation of benzene by the whole fungus (*A*. *aegerita*), will have to be clarified in future physiological studies.

The enzymes characteristics described above suggest a certain application potential of peroxygenases in the preparation of hydroxylated benzene derivatives. Technical synthesis of phenol via the cumene process requires a large energy input and bears several environmental risks (Weber et al. 2000). Thus the environmental friendly production of phenol or further hydroxylated products by an enzymatic process would be a worthwhile approach. Furthermore chemical reactions opening the epoxide-ring of benzene oxide formed by peroxygenase could lead to specifically substituted phenols. Such ambitious goals, however, will necessitate much more research into the basics of peroxygenase catalysis and the heterologous expression of peroxygenases.

## Competing interests

The authors declare that there are no competing interests.
